# A simple circuit to sustain intact tumor microenvironments for complex drug interrogations

**DOI:** 10.1101/2025.10.26.684624

**Published:** 2025-10-28

**Authors:** A Leila Sarvestani, Samantha Ruff, Kirsten Remmert, Martha Teke, Emily Verbus, Areeba Saif, Marcial Garmendia-Cedillos, Alex Rossi, Ashley Rainey, Krystle Nomie, Surajit Sinha, Michael M. Wach, Carrie Ryan, Stephanie N. Gregory, Priyanka Desai, Abir K. Panda, Emily C. Smith, Reed Ayabe, James D. McDonald, Shreya Gupta, Tracey Pu, Tahsin Khan, Jacob Lambdin, Kenneth Luberice, Stephanie C. Lux, Hanna Hong, Imani Alexander, Sarfraz R. Akmal, Shahyan Rehman, Sophia Xiao, Jason Ho, Justine Burke, Cathleen E. Hannah, Alexandra Livanova, Andrey Tyshevich, Stanislav Kurpe, Andrey Kravets, Basim Salem, Daniil Vibe, Arina Tkachukv, Anna Belozerova, Aleksandr Sarachakov, Evgeny Barykin, Vladimir Kushnarev, Ekaterina Postovalova, Alexander Bagaev, Noemi Kedei, Maria Hernandez, Baktiar Karim, Donna Butcher, Jennifer Matta, Michael Kelly, Jatinder Singh, Todd D. Prickett, James Hodge, Thorkell Andresson, Michael B. Yaffe, Ethan M. Shevach, David Kleiner, Jeremy L. Davis, Andrew M. Blakely, Jonathan M. Hernandez

## Abstract

Deep learning and large language models can integrate complex datasets to uncover biological insights that are often undetectable through conventional analyses. With application to translational cancer research, these computational tools have positioned 3D patient-derived tumor avatars front and center as crucial data input sources. However, a major challenge remains: the lack of standardization in media composition in 3D patient-derived tumor models unpredictably affects cell behavior and limit the utility beyond predicting treatment responses. To address this unmet need, we developed a simple, reproducible perfusion circuit system to approximate *in vivo* physiology using autologous patient plasma. With peritoneal metastases and core needle biopsies across multiple tumor histologies, we demonstrate preservation of the tumor microenvironment for up to 48 hours using multi-modal interrogation techniques. With proof-of-concept experiments, we display the system’s ability to unveil complex drug-dependent biology within this time window. Standardizable, physiologically relevant platforms for 3D patient-derived tumor avatars will yield unprecedented insights through the integration of data from broad groups of patients and the use of an expanding armamentarium of artificial intelligence capabilities.

## Full Text Availability

The license terms selected by the author(s) for this preprint version do not permit archiving in PMC. The full text is available from the preprint server.

